# Distinct patterns of progressive gray and white matter degeneration in amyotrophic lateral sclerosis

**DOI:** 10.1002/hbm.25738

**Published:** 2021-12-15

**Authors:** Abdullah Ishaque, Daniel Ta, Muhammad Khan, Lorne Zinman, Lawrence Korngut, Angela Genge, Annie Dionne, Hannah Briemberg, Collin Luk, Yee‐Hong Yang, Christian Beaulieu, Derek Emery, Dean T. Eurich, Richard Frayne, Simon Graham, Alan Wilman, Nicolas Dupré, Sanjay Kalra

**Affiliations:** ^1^ Faculty of Medicine and Dentistry University of Alberta Edmonton Canada; ^2^ Neuroscience and Mental Health Institute University of Alberta Edmonton Canada; ^3^ Division of Neurology, Department of Medicine University of Toronto Toronto Canada; ^4^ Department of Clinical Neurosciences, Hotchkiss Brain Institute University of Calgary Calgary Canada; ^5^ Department of Neurology and Neurosurgery Montreal Neurological Institute Montreal Canada; ^6^ Département des Sciences Neurologiques, Hôpital de l'Enfant‐Jésus, CHU de Québec Quebec City Canada; ^7^ Division of Neurology, Department of Medicine University of British Columbia Vancouver Canada; ^8^ Division of Neurology, Department of Medicine University of Alberta Edmonton Canada; ^9^ Department of Computing Science University of Alberta Edmonton; ^10^ Department of Biomedical Engineering University of Alberta Edmonton Canada; ^11^ Department of Radiology and Diagnostic Imaging University of Alberta Edmonton Canada; ^12^ School of Public Health University of Alberta Edmonton Canada; ^13^ Department of Radiology, Hotchkiss Brain Institute University of Calgary Calgary Canada; ^14^ Seaman Family MR Research Centre Foothills Medical Centre, Alberta Health Services Calgary Canada; ^15^ Department of Medical Biophysics University of Toronto Toronto Canada; ^16^ Neuroscience Axis, CHU de Québec Université Laval Quebec City Canada; ^17^ Department of Medicine, Faculty of Medicine Université Laval Quebec City Canada

## Abstract

Progressive cerebral degeneration in amyotrophic lateral sclerosis (ALS) remains poorly understood. Here, three‐dimensional (3D) texture analysis was used to study longitudinal gray and white matter cerebral degeneration in ALS from routine T1‐weighted magnetic resonance imaging (MRI). Participants were included from the Canadian ALS Neuroimaging Consortium (CALSNIC) who underwent up to three clinical assessments and MRI at four‐month intervals, up to 8 months after baseline (*T*_0_). Three‐dimensional maps of the texture feature autocorrelation were computed from T1‐weighted images. One hundred and nineteen controls and 137 ALS patients were included, with 81 controls and 84 ALS patients returning for at least one follow‐up. At baseline, texture changes in ALS patients were detected in the motor cortex, corticospinal tract, insular cortex, and bilateral frontal and temporal white matter compared to controls. Longitudinal comparison of texture maps between *T*_0_ and *T*_max_ (last follow‐up visit) within ALS patients showed progressive texture alterations in the temporal white matter, insula, and internal capsule. Additionally, when compared to controls, ALS patients had greater texture changes in the frontal and temporal structures at *T*_max_ than at *T*_0_. In subgroup analysis, slow progressing ALS patients had greater progressive texture change in the internal capsule than the fast progressing patients. Contrastingly, fast progressing patients had greater progressive texture changes in the precentral gyrus. These findings suggest that the characteristic longitudinal gray matter pathology in ALS is the progressive involvement of frontotemporal regions rather than a worsening pathology within the motor cortex, and that phenotypic variability is associated with distinct progressive spatial pathology.

## Graphical abstract

In this study, we use MRI texture analysis to detect progressive cerebral degeneration in amyotrophic lateral sclerosis (ALS). Here, we demonstrate distinct spatiotemporal patterns of degeneration related to clinical phenotypes in patients with ALS.

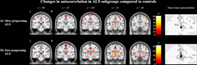

## 1 INTRODUCTION

Amyotrophic lateral sclerosis (ALS) is a rapidly progressive neurodegenerative disease with a median survival of 26 months after diagnosis (Pupillo, Messina, Logroscino, Beghi, & Group, 2014). Its sporadic form typically affects adults between the ages of 60 and 70 years and is characterized by progressive weakness of muscles in the limbs and difficulties with speech and swallowing (Brown & Al‐Chalabi, 2017). Frontotemporal dementia (FTD) is present in approximately 10% of patients at the time of diagnosis, with up to 50% of patients demonstrating cognitive and behavioral deficits on detailed neuropsychometric testing (Phukan et al., 2012). Frontotemporal involvement in ALS is further substantiated by studies demonstrating widespread cortical thinning (Agosta et al., 2012; d'Ambrosio et al., 2014), reductions in gray matter density (Menke et al., 2014), and underlying white matter degeneration (Cirillo et al., 2012; Kasper et al., 2014).

Though the cross‐sectional cerebral neuroimaging signature of ALS is well‐understood as degeneration of the upper motor neuron (UMN) system (including the motor cortex and descending pyramidal tracts) with variable frontotemporal involvement, the progressive degeneration in ALS remains poorly understood. Initial longitudinal studies were limited by small sample sizes (*n* < 20) and a majority of studies have either investigated only gray matter with T1‐weighted images (Floeter et al., 2016; Schuster, Kasper, Machts, et al., 2014; Walhout et al., 2015), or white matter with diffusion tensor imaging (DTI) (Floeter, Danielian, Braun, & Wu, 2018; Kassubek et al., 2018; van der Graaff et al., 2011). However, more comprehensive analyses are required in a disease where both gray and white matter structures are affected. Thus far, only four studies have included more than 20 ALS patients with a multimodal MRI protocol to study whole‐brain progressive changes, though none included more than 35 ALS patients (Bede & Hardiman, 2018; Cardenas‐Blanco et al., 2016; de Albuquerque et al., 2017; Menke et al., 2014). Two of these studies demonstrated progressive changes in the corticospinal tract and found no change in gray matter after 6–8 months (Cardenas‐Blanco et al., 2016; de Albuquerque et al., 2017). In contrast, widespread gray matter degeneration was reported with limited white matter involvement in the other two studies (Bede & Hardiman, 2018; Menke et al., 2014).

Texture analysis is a computational image processing technique that quantifies variations and relationships between voxel intensities in an image, which are difficult to detect by qualitative visual inspection and may not be detectable by common image analysis methods in the field such as voxel‐based morphometry (VBM) and cortical thickness measurements. Autocorrelation is a texture feature that is calculated using the gray‐level co‐occurrence matrix (GLCM), a second‐order texture analysis method (Haralick, Shanmugam, & Dinstein, 1973). It is sensitive to the absolute intensity, or gray level, of voxels and is a measure of co‐occurring voxel intensities. Two‐dimensional (2D) texture analysis methods have been utilized extensively in other neurological conditions such as brain tumors, stroke, epilepsy, and multiple sclerosis to detect and classify lesions (Kassner & Thornhill, 2010). Our group developed a 3D extension of GLCM to enable whole‐brain voxel‐wise analysis of texture features (Maani, Yang, & Kalra, 2015). With this technique, we showed that autocorrelation calculated from T1‐weighted images is altered in ALS compared to controls in regions of the motor cortex, frontal lobe, temporal lobe, and posterior limb of the internal capsule (PLIC) (Ishaque, Mah, Seres, Luk, Eurich, et al., 2018; Maani, Yang, Emery, & Kalra, 2016). This pattern of cerebral change is in agreement with previously published structural imaging studies in ALS (Li et al., 2012; Shen et al., 2016). Furthermore, alterations in autocorrelation in the corticospinal tract on T1‐weighted images in ALS are related to abnormalities observed with DTI metrics (Ishaque, Mah, Seres, Luk, Johnston, et al., 2018). Texture‐based abnormalities in T1‐weighted images can therefore successfully recapitulate the known gray and white matter pathology in ALS.

A comprehensive evaluation of progressive cerebral degeneration in ALS is critical to further the understanding of the pathophysiology of the disease. As such, the primary objectives of this study were to (1) examine the cerebral changes in patients with ALS over an 8‐month period with texture analysis of T1‐weighted images, and (2) to evaluate whether the progressive changes are different between slow and fast progressing patients. Patterns of longitudinal change and the relationships between functional disability, extent of UMN involvement, and texture were also assessed as secondary objectives. The study design included whole‐brain and region‐of‐interest (ROI)‐based approaches to investigate the changes in texture. We hypothesized that (1) texture alterations in T1‐weighted images are present in gray and white matter and associated with the known pathology and clinical impairment in ALS; (2) progressive cerebral degeneration is evident as texture alterations over time; and (3) progressive cerebral changes in fast progressing patients are greater than the changes in slow progressing patients. To test our hypotheses, we conducted the study in a large, multicentre cohort of ALS patients and controls.

## 2 MATERIALS AND METHODS

### 2.1 Participants

Participants for this study were prospectively recruited from six different ALS clinics as part of the Canadian ALS Neuroimaging Consortium (CALSNIC), a multicentre research platform for biomarker (Kalra et al., 2019). The CALSNIC research protocol consists of clinical assessments and MRI scans at baseline (*T*_0_), four (*T*_4_) and 8 months (*T*_8_); data from all available timepoints were used. *T*_max_ is defined as the last follow‐up visit attended by the participant. Patients were included in this study if they had signs of UMN and lower motor neuron (LMN) dysfunction in at least one body region on neurological examination and had a diagnosis of possible, probable, probable laboratory supported, or definite ALS as defined by the El Escorial criteria (Brooks, Miller, Swash, & Munsat, 2000). Patients with a family history of ALS and/or FTD, a causative genetic mutation, or comorbid FTD were included. Diagnoses of ALS‐FTD were made by trained neurologists specializing in ALS with formal neuropsychological evaluation prior to participant recruitment. Patients were excluded if they had a known history of any other neurological or psychiatric disorders. Control participants with no known neurological or psychiatric disorders were recruited from each site. Institutional ethics approvals were obtained from all recruiting centers and participants provided free and informed written consent prior to their involvement.

### 2.2 Clinical assessment

Patient functional disability was assessed at all timepoints using the ALS Functional Rating Scale‐Revised (ALSFRS‐R). ALSFRS‐R is a 48‐point questionnaire that quantifies patient disability related to bulbar, limb, axial, and respiratory function with lower scores representing increased disability (Cedarbaum et al., 1999). Symptom duration was calculated as the time in months from symptom onset to the day the ALSFRS‐R was administered. Disease progression rate was quantified as (48 − ALSFRS‐R score)/symptom duration. Patients underwent a clinical neurological examination of their muscle tone and reflexes at all timepoints by neurologists specializing in ALS. Based on the clinical exam, each patient was assigned a UMN burden score out of 16 where higher scores indicate greater UMN dysfunction; the calculation of the score is provided in Table S1. Incomplete neurological exams were excluded from analyses. Bilateral finger and foot tapping scores were measured for each participant at all timepoints. Participants were instructed to tap their finger and foot as fast as possible over a 1‐min period for two trials. The final finger and foot tapping scores were calculated as the means of their bilateral scores over the two trials. Tapping scores of 0 were excluded from analyses. The Edinburgh Cognitive and Behavioral ALS Screen (ECAS) is a comprehensive screening tool used to evaluate cognitive domains of language, verbal fluency, memory, visuospatial, and executive functioning. It was collected for each participant in the study sample. Using this tool, cognitive impairment was defined using a previously defined cut‐off score of 105 out of a total of 136 (Abrahams, Newton, Elaine, Foley, & Bak, 2014).

### 2.3 Magnetic resonance imaging

3D T1‐weighted images were acquired at 1 mm isotropic resolution at all timepoints on 3T MRI systems as part of the larger CALSNIC MRI protocol (Kalra et al., 2019). Table S2 details the MRI acquisition parameters at each of the six recruiting centers.

### 2.4 Image processing

Image processing and analyses were carried out in Statistical Parametric Mapping 12 (SPM12) (https://www.fil.ion.ucl.ac.uk/spm/) and the Computational Anatomy Toolbox 12 (http://www.neuro.uni-jena.de/cat/) software. All T1‐weighted images were aligned along the anterior commissure‐poster commissure line for optimal image processing and texture analysis. These were then processed using the CAT12 “Segment Data” pipeline where they underwent bias field correction, segmentation into gray and white matter tissue classes, and normalization to the supplied Montreal Neurological Institute (MNI) template using the Diffeomorphic Anatomical Registration Through Exponentiated Lie Algebra (DARTEL) approach at default settings (Ashburner, 2007). Deformation field maps for the native‐to‐standard space image transformation were saved for each participant and their individual timepoints.

The 3D GLCM texture analysis was performed using a toolbox developed for SPM (Maani et al., 2015). For texture analysis, the average duration of processing was approximately 15 min per T1‐weighted image using a PC with an Intel Quad Core 3.40 GHz CPU with 16gb RAM on Windows 10 Profession using MATLAB 2018a. Whole‐brain maps for autocorrelation were calculated from the bias‐corrected T1‐weighted volume images in their native space. Autocorrelation quantifies the linear dependency and repetitive patterns in pairs of gray levels in a local neighborhood of voxels in an image. Further technical details describing its calculation have been published previously (Ishaque, Mah, Seres, Luk, Eurich, et al., 2018; Maani et al., 2015). The saved deformation field map for each T1‐weighted image was applied to its respective autocorrelation map to transform the map to the MNI space. The transformed autocorrelation maps were smoothed with a 6 mm full‐width at half‐maximum Gaussian kernel prior to voxel‐wise analyses.

The voxel‐wise analyses allowed investigations of whole‐brain group comparisons and clinical correlations in an unbiased manner. Combined with texture analysis of T1‐weighted images, this approach enabled the assessments of both gray and white matter structures without a priori hypotheses. In addition, ROI‐based analyses of the motor structures were also performed to probe their specific clinical correlations and to quantify their longitudinal progression in ALS. The ROI‐based analyses also served a secondary purpose of verifying the results of voxel‐wise analyses. Mean autocorrelation values from the precentral gyrus and the PLIC were extracted. The mask for the bilateral precentral gyrus was obtained from the Harvard‐Oxford cortical structural atlas at a 25% threshold and the mask for the PLIC was obtained from the Johns Hopkins University white matter label atlas (Mori et al., 2008). These masks were applied to the unsmoothed autocorrelation maps in the MNI space and their mean autocorrelation value for each region was calculated. This was done for data from all time points.

### 2.5 Statistical analysis

Quantitative demographic, clinical, and ROI data were analyzed in MedCalc Statistical Software version 19.1.3 (MedCalc Software bvba, Ostend, Belgium; https://www.medcalc.org; 2019). Results are presented as mean ± SD unless otherwise stated. Between‐group differences were assessed with independent samples *t*‐tests, *χ* ^2^ tests, and analysis of covariance where appropriate. Pearson correlation coefficient, *r*, was used to test for associations among clinical variables and autocorrelation. Corrections for multiple comparisons were not employed because the testing of individual clinical associations was hypothesis‐driven. Patients were classified as “slow progressing” if their disease progression rate was lower than the patient group's median rate, or as “fast progressing” if it was higher. Statistical significance was defined at *p* < .05.

Voxel‐wise analyses were conducted in SPM12. Full‐factorial models were used to assess between‐group, whole‐brain differences in autocorrelation. The models included “group” as the variable of interest and age as a covariate. Site was not included as a factor because autocorrelation had previously been shown to have high intra‐ and intersite reliability in voxel‐wise and ROI‐based analyses in an intraclass correlation coefficient study (Ta et al., 2019). As an added measure, the main voxel‐wise group analysis was also performed with and without a correctional factor for site (data not shown). The results from both of these models were nearly identical, and thus, site was omitted as a factor for all analyses in favor of simplicity. A *T*‐contrast was used to establish the directionality of change in autocorrelation (increased or decreased) in ALS compared to controls. *F*‐contrasts were subsequently used to test for the absolute changes in autocorrelation between groups. The following clinical measures were tested as variables of interest at *T*_0_: (1) ALSFRS‐R score, (2) UMN burden score, (3) average finger tapping score, and (4) average foot tapping score. Whole‐brain voxel‐wise paired *t*‐tests were conducted in patients to compare their autocorrelation maps at the different timepoints. Regression models were used to assess for whole‐brain associations between autocorrelation and clinical variables in ALS patients. Significant clusters were identified at *p* < .0005 with a minimum cluster size of at least 50 voxels for all voxel‐wise analyses (Chen et al., 2018; Sheng et al., 2015). Voxel‐wise analyses were corrected for age as it was added as a covariate for all statistical models.

Linear mixed‐effects models were used to assess the longitudinal progression of clinical measures and autocorrelation in the ROIs in ALS patients. The time interval from *T*_0_ in months was used as the fixed effect. The model intercept and the time interval were used as the random effects. Longitudinal change in autocorrelation was additionally investigated in the ALS subgroups and controls. The interaction between group assignment and monthly decline in autocorrelation was also tested between ALS and controls, and slow and fast progressing ALS. All linear mixed‐effect models were analyzed in SPSS (IBM Corp. Released 2016. IBM SPSS Statistics for Windows, Version 20.0. Armonk, NY: IBM Corp.). Statistical significance was defined at *p* < .05.

## 3 RESULTS

### 3.1 Study sample characteristics

A total of 256 participants (119 controls and 137 ALS patients) met the inclusion criteria for this study (Table 1). The mean age of ALS patients was higher than controls (*p* = .02), and there were proportionally more males than females in the ALS group than in the control group (*p* = .04). The mean ALSFRS‐R score was 37.8 ± 5.7 (*n* = 134), UMN burden was 5.4 ± 2.9 (*n* = 125), and finger and foot tapping scores were 43.3 ± 13.2 (*n* = 94) and 29.0 ± 12.2 (*n* = 80), respectively. The median disease progression rate for ALS patients was 0.4 (range 0.02–2.1) and this was used to divide the patients into slow and fast progressing ALS subgroups. Participants in these two subgroups had no differences in their mean age and gender distribution (*p* = .4 and .5, respectively; Table S3). Participants in the fast progressing ALS subgroup had a higher mean UMN burden score (*p* = .002) and a greater proportion of patients with bulbar‐onset ALS (*p* = .004). Out of 137 patients with ALS, 50 patients had a total ECAS score below 105, representing 36% of the total ALS cohort. Further clinical and demographic details for ALS subgroups are provided in Table S3.

**TABLE 1:** Baseline characteristics of study participants

*n*	Controls	ALS	*p* value
	119	137	
Age (years)	55.8 ± 10.4	59.1 ± 10.5	.01^a^
Gender, male/female	58/61	85/52	.03^b^
Site of symptom onset, limb/bulbar	—	110/27	
ALSFRS‐R	—	39 (20–47)	
Symptom duration (months)	—	23.4 (5.7–151.7)	
Disease progression rate	—	0.4 (0.02–2.1)	
UMN burden score	—	5 (1–12)	
Average finger tapping score	59.0 ± 12.2	43.3 ± 13.2	<.001^a^
Average foot tapping score	43.4 ± 8.5	29.0 ± 12.2	<.001^a^

*Note*: Data are represented as mean ± *SD*, or median (range) if data did not follow a normal distribution (Shapiro–Wilk test, *p* < .05). Significant between‐group differences: *p* < .05.

Abbreviations: ALSFRS‐R, amyotrophic lateral sclerosis functional rating scale‐revised; disease progression rate = (48 − ALSFRS‐R)/symptom duration; UMN, upper motor neuron.

^a^ Independent samples *t*‐test.

^b^ Chi‐squared test.

Eighty‐one controls and 84 ALS patients returned for at least one follow‐up MRI scan at *T*_4_. Fifty‐seven controls and 49 ALS patients returned for follow‐up scans at *T*_4_ and *T*_8_. A total of 528 MRI datasets from ALS patients and controls were therefore included in this study (*T*_0_ = 257, *T*_4_ = 159, *T*_8_ = 112). At the time of analysis, not all participants had reached their *T*_4_ and/or *T*_8_ assessments and therefore an attrition rate was not determined. The mean time from *T*_0_ to *T*_max_ was 243.9 ± 74.6 days and 212.8 ± 68.2 days for controls and ALS patients, respectively. There were no differences in the mean age and gender distribution between ALS patients who returned for a follow‐up and those who did not (*p* = .8 and .4, respectively). The mean UMN burden score at baseline also did not differ between these two groups (*p* = .7). ALS patients who returned for a follow‐up had a higher mean ALSFRS‐R score at *T*_0_ (39.0 ± 4.8) compared to those who did not return (35.6 ± 6.5; *p* < .001); however, there was no significant difference between the two groups' disease progression rates (0.4 ± 0.4 versus 0.5 ± 0.4, respectively; *p* = .1). Of the 110 patients with limb‐onset ALS at baseline, 74 (67.2%) returned for at least one follow‐up MRI scan. In contrast, 10 of the 27 (37.0%) patients with bulbar‐onset ALS returned for a follow‐up (*p* = .004).

### 3.2 Group differences in texture at baseline

**FIGURE 1:**
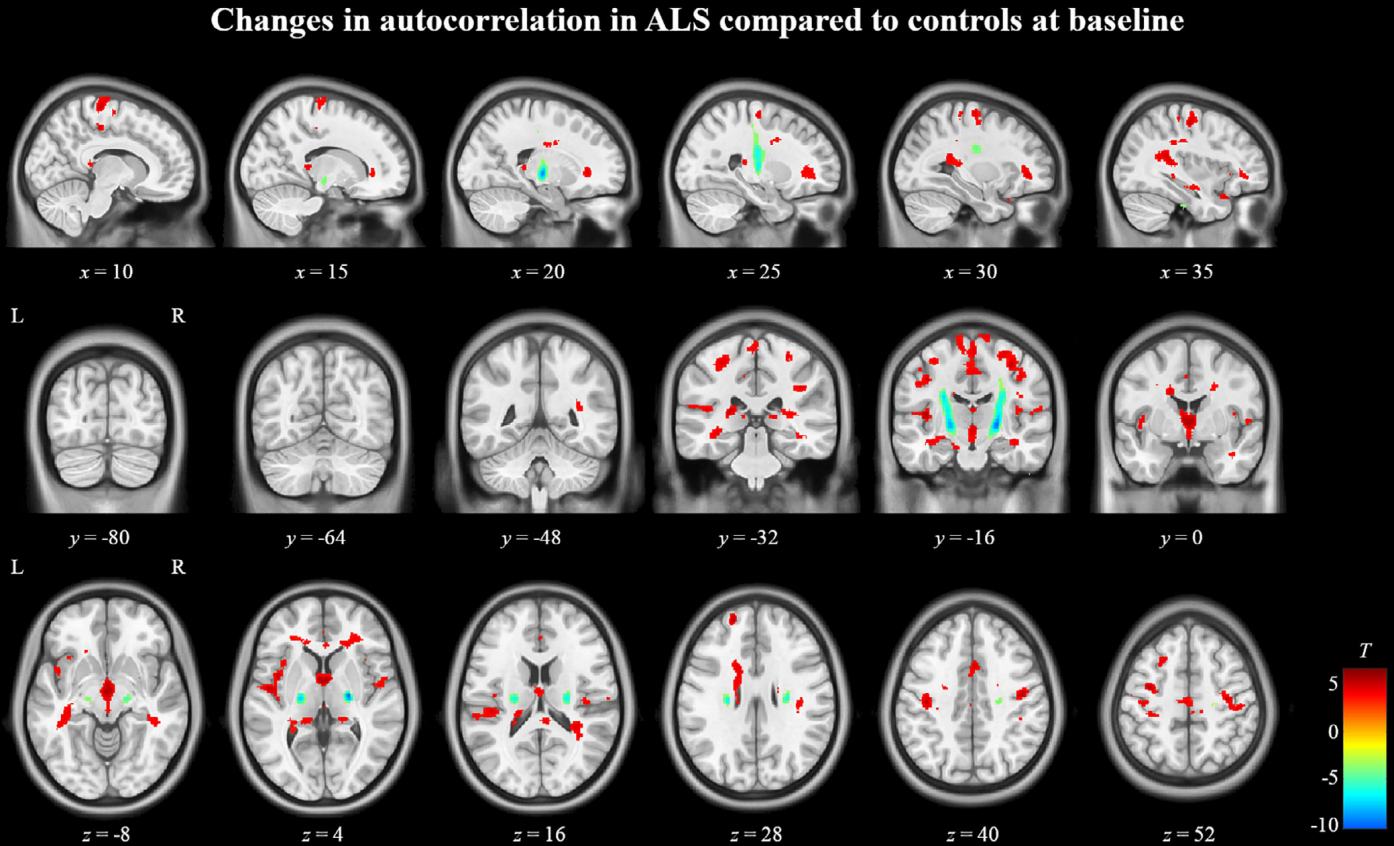
Texture differences between ALS patients and controls at *T*_0_. Regions in red indicate areas of significantly (*p* < .0005, cluster size >50) decreased autocorrelation in ALS patients and regions in blue indicate areas of significantly increased autocorrelation. The color bar on the bottom right shows the range of *T*‐values for the contrast controls >ALS patients

In whole‐brain group comparison, ALS patients had decreased autocorrelation compared to controls in bilateral precentral gyri, subcortical white matter, left supplementary motor area, left frontal middle and superior gyri, bilateral frontal white matter, bilateral insular cortex, and bilateral temporal white matter (Figure 1). Autocorrelation was increased in ALS patients along bilateral pyramidal tracts in regions between the corona radiata and the cerebral peduncles (Figure 1). Significant clusters in bilateral pyramidal tracts and medial precentral gyrus were present even after the application of progressively restrictive statistical thresholds of *p* < .00005, .000005, and .0000005 (Figure S1). Clusters in the left insular cortex and thalamic region appeared at *p* < .000005, followed by clusters in the temporal lobe at the lowest statistical threshold of *p* < .00005.

**FIGURE 2:**
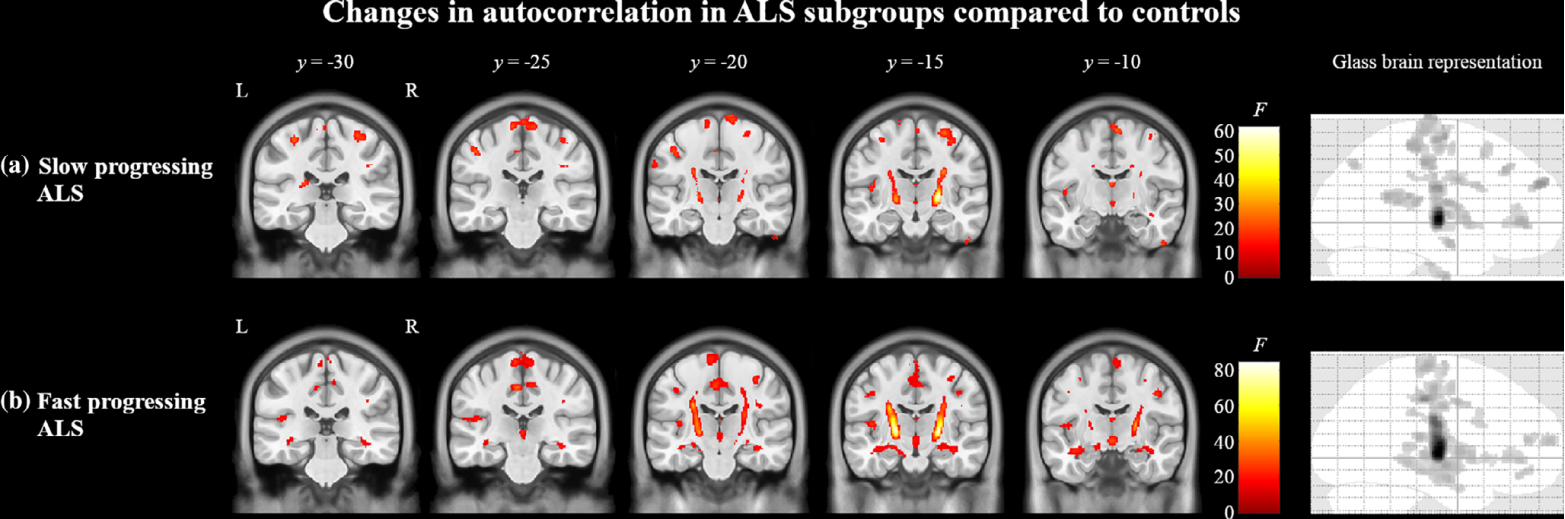
Texture differences between ALS subgroups of slow and fast progressing patients compared to controls at *T*_0_. In panel (a), regions in red indicate areas of significantly (*p* < .0005, cluster size >50) altered autocorrelation in slow progressing ALS patients compared to controls. In panel (b), regions in red indicate areas of significantly altered autocorrelation in fast progressing ALS patients compared to controls. The color bars show the range of *F*‐values

Compared to controls, the slow progressing ALS group had alterations in autocorrelation in bilateral precentral gyri, left middle frontal gyrus, bilateral frontal white matter, left insular cortex, and bilateral pyramidal tracts (Figure 2a). In contrast, the fast progressing ALS group had fewer regions of altered autocorrelation in the frontal cortex, but a greater involvement of bilateral pyramidal tracts, temporal white matter, and parahippocampal regions (Figure 2b). There were no significant differences between the subgroups when compared directly.

In the ROI‐based analysis, autocorrelation was decreased in ALS patients in the bilateral precentral gyrus (estimated marginal mean ± SE: 42.4 ± 0.2) compared to controls (43.1 ± 0.2) when covaried with age (*p* = .004). Similarly, in the PLIC ROI, autocorrelation was significantly (*p* < .001) increased in ALS patients (82.9 ± 0.2) compared to controls (81.2 ± 0.2). No significant difference was present in autocorrelation between the subgroups in the precentral gyrus (slow progressing ALS: 42.3 ± 0.3 and fast progressing ALS: 42.6 ± 0.3; *p* = .3) or in the PLIC (slow progressing ALS: 82.6 ± 0.3 and fast progressing ALS: 83.2 ± 0.3; *p* = .1).

### 3.3 Longitudinal changes in texture

**FIGURE 3:**
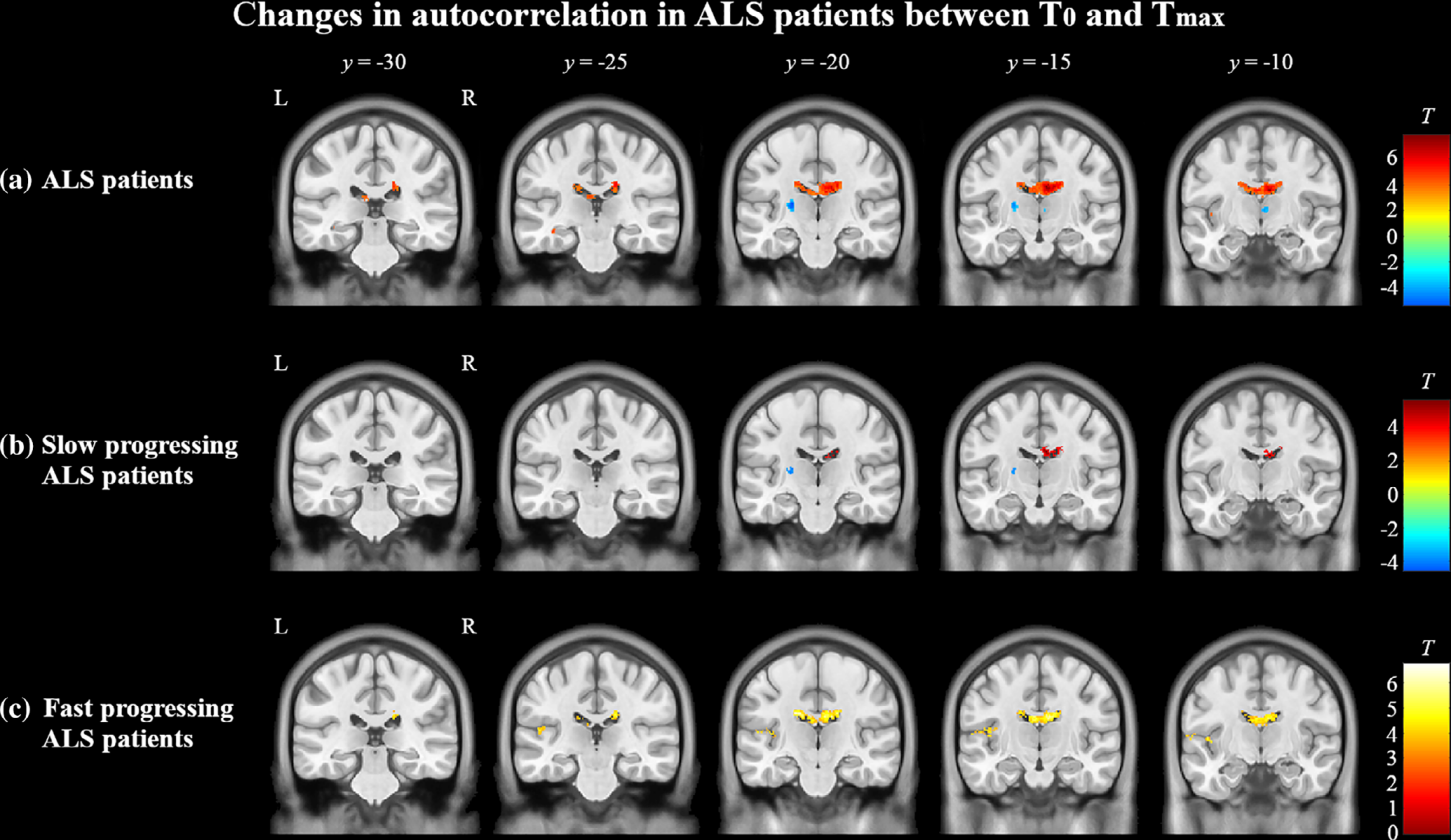
Longitudinal changes in texture in (a) ALS patients and (b) slow and (c) fast progressing subgroups from *T*_0_ to *T*_max_ evaluated using paired *t*‐tests (*p* < .0005, cluster size >50). (a) In all ALS patients, autocorrelation decreased longitudinally (red) in the posterior corpus callosum, left insular cortex, and at the junction of lateral ventricles and bilateral caudate heads. Autocorrelation increased (blue) in the left internal capsule and right thalamus (*p* < .0005, cluster size >50). (b) In slow progressing ALS, autocorrelation decreased in the posterior corpus callosum and increased in the left internal capsule. (c) In fast progressing ALS, autocorrelation decreased in the posterior corpus callosum and left insular cortex. Autocorrelation was not increased in this subgroup. The color bars show the range of *T*‐values

In the whole‐brain paired *t*‐test comparing ALS patients at *T*_0_ and *T*_max_ (*n* = 84), autocorrelation was significantly decreased at *T*_max_ in the posterior corpus callosum, left insular cortex, left temporal white matter, and along the junction of lateral ventricles and bilateral caudate heads compared to *T*_0_ (Figure 3a). Additionally, autocorrelation was significantly increased in the left internal capsule and right thalamus at *T*_max_ (Figure 3a). In the slow progressing ALS subgroup with at least one follow‐up (*n* = 44), autocorrelation was significantly decreased in the posterior corpus callosum and significantly increased in the left internal capsule at *T*_max_ compared to *T*_0_ (Figure 3b). In contrast, autocorrelation was significantly decreased in the posterior corpus callosum, left insular cortex, and at the junction of lateral ventricles and bilateral caudate heads in fast progressing ALS (*n* = 40) at *T*_max_ compared to *T*_0_ (Figure 3c). No areas were significantly increased in this ALS subgroup. No whole‐brain differences were found with paired *t*‐test between controls at *T*_0_ and *T*_max_.

Progressive changes between all three timepoints were assessed with whole‐brain paired *t*‐tests in the subset of ALS patients who returned for all timepoints (*n* = 49). Between *T*_0_ and *T*_4_, autocorrelation was significantly decreased in the posterior corpus callosum (Figure S2A). Between *T*_0_ and *T*_8_, autocorrelation was significantly decreased in the posterior corpus callosum and along the junction of lateral ventricles and bilateral caudate heads (Figure S2B). Autocorrelation was also significantly increased in the right thalamus in this comparison.

**FIGURE 4:**
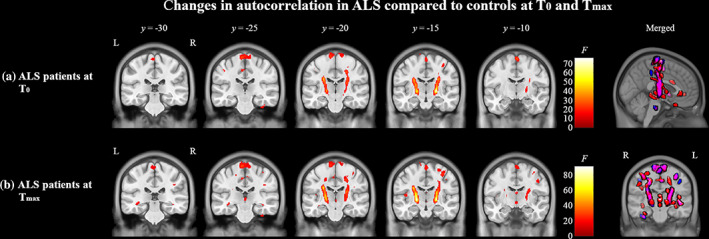
Texture differences between ALS compared to controls at *T*_0_ (a) and *T*_max_ (b). In panels (a) and (b), regions in red indicate areas of significantly (*p* < .0005, cluster size >50) altered autocorrelation in ALS patients compared to controls. The color bars show the range of *F*‐values. Images on the right show a merged glass‐brain representation of the differences in ALS patients at *T*_0_ and *T*_max_. Regions in blue indicate significant clusters present only at *T*_0_, regions in purple indicate significant overlapping clusters present at *T*_0_ and *T*_max_, and regions in red indicate significant clusters present only at *T*_max_

Longitudinal changes in texture in ALS were also assessed with group analyses between ALS patients and controls at *T*_0_ and *T*_max_. At *T*_0_, ALS patients had alterations in autocorrelation in the precentral gyrus, bilateral pyramidal tracts, and left insular cortex compared to controls when covaried with age (Figure 4a). At *T*_max_, in addition to the changes observed at *T*_0_, these ALS patients had further alterations in autocorrelation in the frontal lobe white matter, bilateral temporal lobe white matter hippocampus, and thalamus compared to controls (Figure 4b). Furthermore, there was an increase in the size of the significant clusters at *T*_max_ compared to *T*_0_.

Linear mixed models were used to investigate the longitudinal evolution of autocorrelation in the precentral gyrus and PLIC ROIs. Controls demonstrated no significant longitudinal change in autocorrelation in either the precentral gyrus (*p* = .7) or the PLIC (*p* = .5). In ALS patients, there was no significant decline in autocorrelation within the precentral gyrus ROI over time (0.005 ± 0.009 [SE] unit/month, *p* = .4). Autocorrelation in the PLIC increased significantly at a rate of 0.05 ± 0.02 (SE) unit/month (*p* = .003). When compared directly, the rate of change in autocorrelation over time was significantly different between ALS patients and controls in the PLIC (*p* = .008), but not in the precentral gyrus (*p* = .9).

**FIGURE 5:**
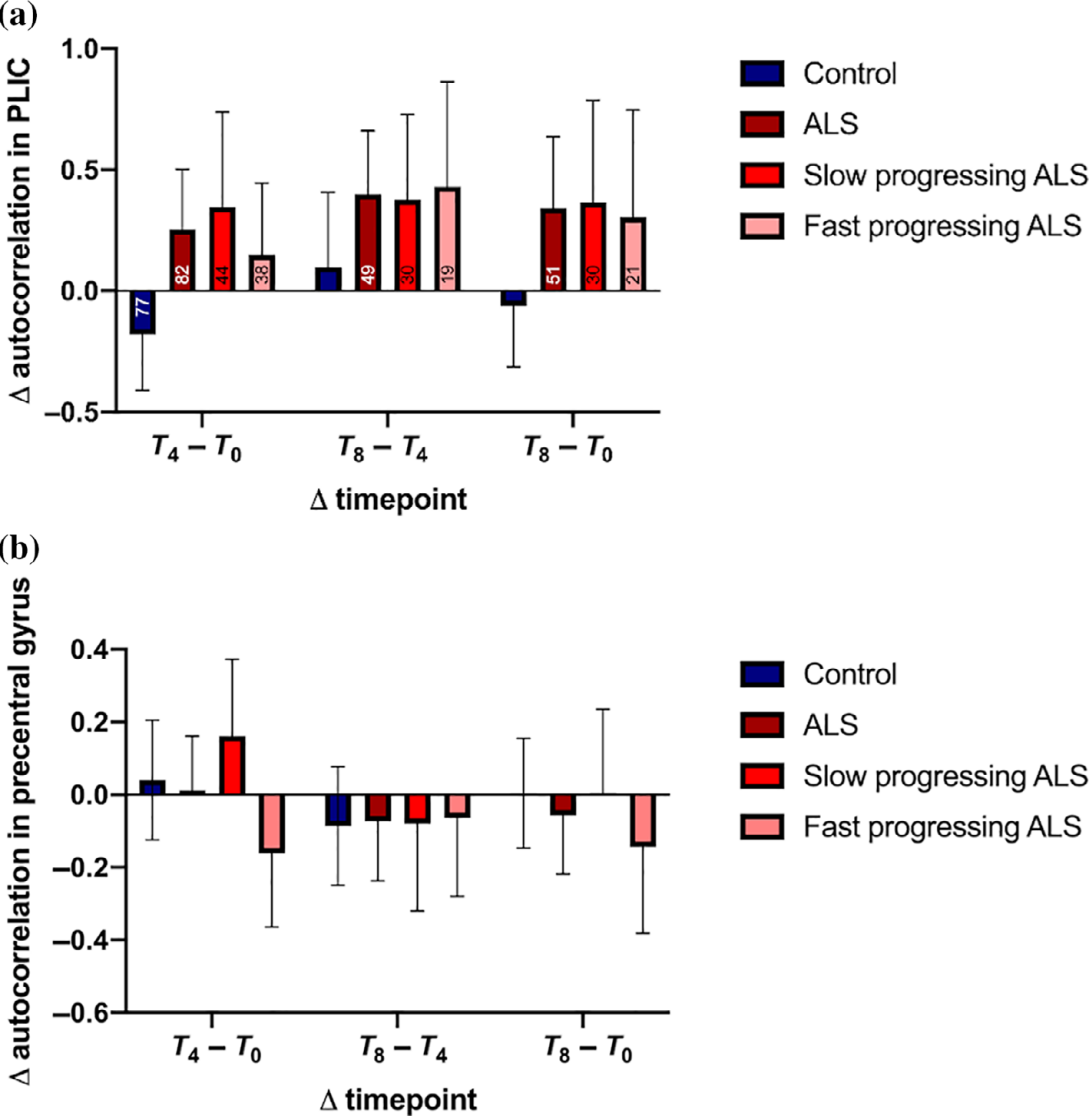
Texture differences in controls, ALS, and ALS subgroups between various timepoints in (a) the PLIC and (b) the precentral gyrus regions of interests. Data are represented as the mean ± 95% CI at each point. The numbers inside in the bars in (a) represent the sample sizes of each group in the respective analyses

In the fast progressing ALS subgroup, there was a trend toward a significant monthly decline in autocorrelation in the precentral gyrus of 0.03 ± 0.01 unit (*p* = .08). In the slow progressing ALS subgroup, there was no significant change over time in autocorrelation in the precentral gyrus (0.007 ± 0.01 unit/month, *p* = .5). There was a trend toward a significant difference between fast and slow progressing ALS in their rates of change in autocorrelation in the precentral gyrus (*p* = .09). In slow progressing ALS, there was a significant increase of 0.06 ± 0.02 unit/month (*p* = .02) in the PLIC. The rate of change increase in autocorrelation (0.05 ± 0.03 unit) did not reach significance (*p* = .08) in fast progressing ALS. When compared directly, there was no significant difference between the two subgroups in their rates of change in autocorrelation over time in the PLIC (*p* = .6). Figure 5 shows the change in autocorrelation in the precentral gyrus and the PLIC between all timepoints in controls, ALS patients, and ALS subgroups.

### 3.4 Clinical measures: Correlations and longitudinal changes

ALSFRS‐R scores correlated with UMN burden scores (*r* = −0.2, *p* = .01), and finger (*r* = 0.5, *p* < .001) and foot tapping scores (*r* = 0.4, *p* < .001; Figure S3). UMN burden scores correlated with finger (*r* = −0.4, *p* < .001) and foot tapping scores (*r* = −0.4, *p* < .001; Figure S3). The longitudinal decline in clinical measures was assessed by linear mixed‐effect models. An average monthly decline of 0.5 ± 0.06 (SE) points was observed in the ALSFRS‐R score (*p* < .001; Figure S4). UMN burden scores did not demonstrate a significant longitudinal monthly change (0.2 ± 0.03, *p* = .6). Finger (0.6 ± 0.2, *p* < .001) and foot tapping scores (0.5 ± 0.1, *p* = .001) also demonstrated significant monthly declines in ALS patients (Figure S4).

### 3.5 Clinical measures: Associations with texture

**FIGURE 6:**
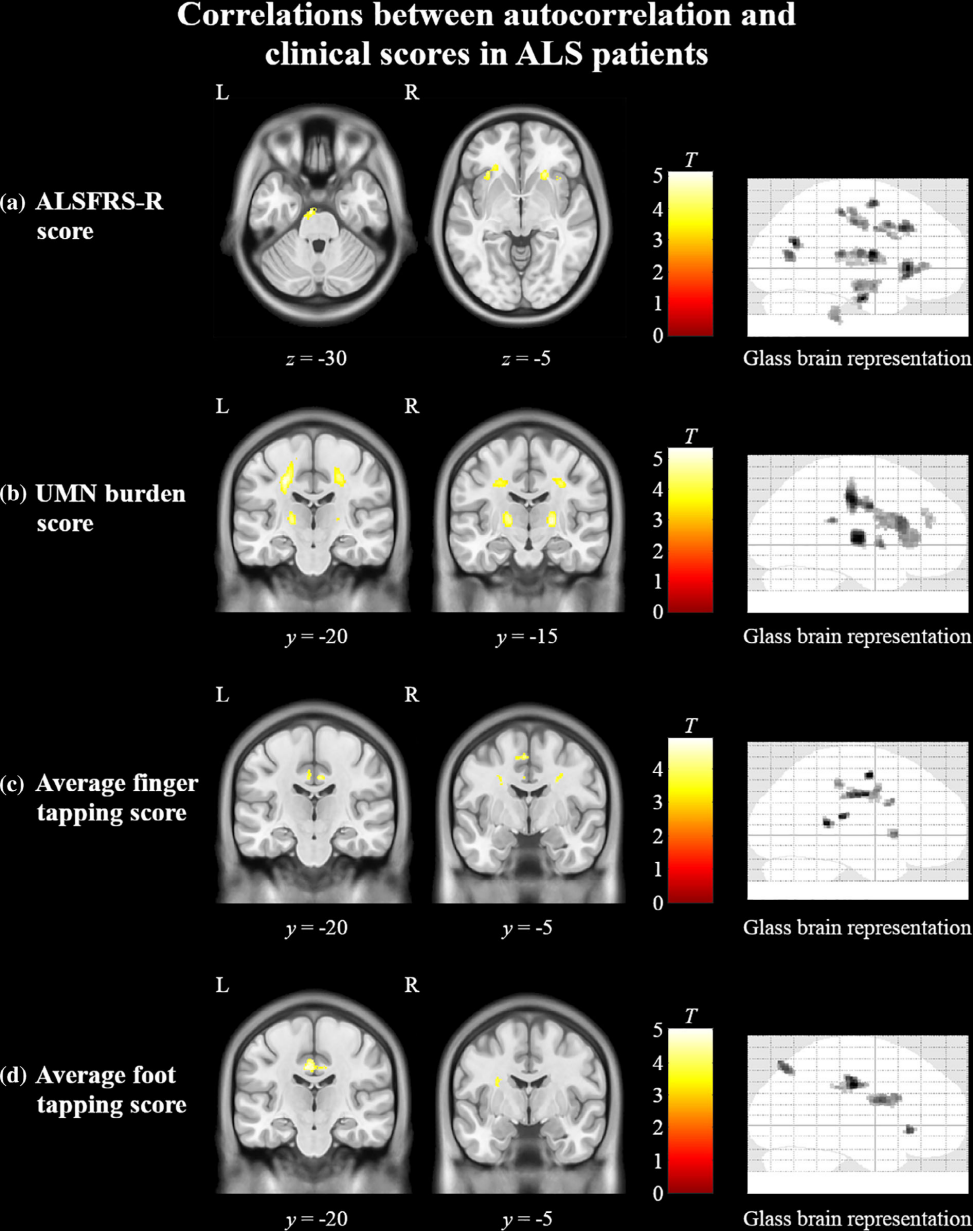
Cerebral associations between texture and clinical measures in ALS patients. Regions in yellow indicate areas of significant (*p* < .0005, cluster size >50) positive correlations between autocorrelation and (a) ALSFRS‐R, (b) UMN burden score, (c) average finger tapping score, and (d) average foot tapping score. The color bars show the range of T‐values. ALSFRS‐R, ALS functional rate scale‐revised; UMN, upper motor neuron

In whole‐brain analysis, the ALSFRS‐R displayed widespread positive correlations with autocorrelation in the white matter regions of the frontal lobe, right insula, right precentral gyrus, left postcentral gyrus, bilateral hippocampal and parahippocampal regions, and in the pons of the brainstem (Figure 6a). Positive correlations between UMN burden score and autocorrelation localized along the bilateral pyramidal tracts in the corona radiata and the internal capsule (Figure 6b). Additional associations with UMN burden were seen in bilateral caudate head. Finger tapping positively correlated with autocorrelation in ALS patients in the frontal lobe white matter, right middle precentral gyrus, left supplementary motor lobule, and bilateral posterior cingulate gyrus (Figure 6c). Foot tapping scores demonstrated positive correlations in the frontal lobe white matter and bilateral posterior cingulate gyrus (Figure 6d).

Autocorrelation from the precentral gyrus ROI significantly correlated with the average finger tapping score (*r* = 0.3, *p* = .003). Autocorrelation from the PLIC significantly correlated with UMN burden score (*r* = 0.3, *p* = .002; Figure S5). There were no other significant correlations between autocorrelation and clinical measures.

## 4 DISCUSSION

In this study, we set out to investigate progressive cerebral degeneration in ALS with texture analysis of T1‐weighted images in a large, multicentre cohort. We first showed that texture‐based abnormalities in gray and white matter at baseline were spatially congruent with the cerebral pathology of ALS. Importantly, texture alterations in the pyramidal tract were also found to be highly specific for clinical UMN dysfunction. This was in contrast to ALSFRS‐R and finger and foot tapping scores that showed diffuse associations to gray and white matter structures. Furthermore, longitudinal analyses revealed that gray matter progression was characterized by spread of pathology toward the frontotemporal regions. We observed progressive changes in the pyramidal tracts after only 4 months. This is a novel observation and of importance as clinical UMN dysfunction did not progress over this time. Lastly, we showed that progressive cerebral degeneration in ALS was predicated upon the disease progression rate at baseline. Taken together, these findings also strongly suggest that texture analysis of T1‐weighted images is a sensitive marker for longitudinal mapping of disease‐related cerebral degeneration in ALS.

### 4.1 Progression of cerebral degeneration in ALS

Though the mechanisms by which progressive cerebral pathology in ALS is disseminated are far from clear, propagation of misfolded proteins via a “prion‐like” mechanism is a leading hypothesis (Polymenidou & Cleveland, 2011). Pathological proteins TDP‐43 and SOD1 in ALS form seeding aggregates (Johnson et al., 2009; Watanabe et al., 2001) that are believed to propagate via axonal pathways between connected regions leading to a stereotyped spread of disease in the brain (Jucker & Walker, 2013). The four stages of TDP‐43 pathology demonstrate a frontotemporal pattern of dissemination with involvement of prefrontal structures in stage 3 and temporal structures in stage 4 (Brettschneider et al., 2013). Therefore, it is reasonable to hypothesize that longitudinal degeneration in ALS should demonstrate a progressive involvement of frontotemporal structures. In our study, greater texture abnormalities were noted in the frontotemporal regions, insula, and subcortical structures. Insular dysfunction has been previously identified as a contributing factor to deficits in verbal fluency (Abrahams et al., 1996), representing a form of executive dysfunction in ALS (Abrahams et al., 2000). As such, progressive involvement of insular structures may contribute to the overall cognitive decline in patients with ALS, though further clinico‐radiological investigations are required to validate this relationship. Atrophic changes in the insula have also shown associations with impaired cognitive flexibility in ALS (Evans et al., 2015), and abnormal TDP‐43 deposition in the insula is observed in 26–49% of patients (Cykowski et al., 2017). Furthermore, recent structural imaging studies have shown progressive atrophy localized to subcortical structures, such as the thalamus and basal ganglia (Bede et al., 2013b; Finegan et al., 2020; Menke, Proudfoot, Talbot, & Turner, 2017; van der Burgh et al., 2020). Texture analysis in susceptibility‐weighted images (SWI) have also demonstrated alterations in the thalamus and basal ganglia, suggesting disturbed iron metabolism within these structures (Johns et al., 2019). Indeed, these abnormalities have been linked with clinical function, with greater atrophy correlating with greater cognitive and motor deficits (Machts et al., 2015; Sharma, Sheriff, Maudsley, & Govind, 2013). Taken together, there is considerable evidence for longitudinal degeneration within subcortical structures, which may be a viable biomarker for disease progression in ALS. Interestingly, we did not find longitudinal change in the precentral gyrus to be different between ALS patients and controls in ROI‐based analysis. Indeed, studies investigating longitudinal cortical thickness have demonstrated thinning of the frontal and temporal cortices with sparing of the precentral gyrus (Schuster, Kasper, Dyrba, et al., 2014; Verstraete et al., 2012; Walhout et al., 2015). This has clinical implications as we previously demonstrated that patients with a shorter survival have greater texture abnormalities in the frontotemporal and insular regions with relatively comparable degeneration of motor cortex compared to patients with a longer survival (Ishaque, Mah, Seres, Luk, Eurich, et al., 2018). Taken together, these findings suggest that further degeneration within the motor cortex in ALS is limited after a critical level of damage is reached. As such, targeted therapies may play a disease‐modifying role if they can halt the disseminative pathology within the gray matter. Future studies should monitor gray matter changes in ALS by assessing the involvement of extra‐motor frontal and temporal structures in a staged manner over time, akin to the proposed DTI‐based staging system (Kassubek et al., 2014; Kassubek et al., 2018).

Pathology of the pyramidal tracts in ALS is believed to be well‐established by the time of diagnosis with limited subsequent longitudinal progression (Bede & Hardiman, 2018; Menke et al., 2014). In particular, a recent study found no change in the pyramidal tracts after 4 months with DTI analysis (Bede & Hardiman, 2018). In the current study, however, progressive degeneration of the pyramidal tracts was detected at 4 months. Strikingly, we did not detect concurrent progressive decline in clinical UMN dysfunction, which is also in agreement with previously published data (Menke et al., 2014). This suggests that texture abnormalities within the pyramidal tracts can monitor subclinical UMN dysfunction and importantly, provide a much‐needed marker for ALS. A formal comparison is needed to ascertain whether or not alterations in texture are more sensitive than DTI in detecting longitudinal degenerative changes.

We also noted abnormalities in texture in the corpus callosum in ALS patients over time. Corpus callosum degeneration is a key component of the cross‐sectional DTI signature in ALS (Filippini et al., 2010). Longitudinal changes in the corpus callosum have also been noted in other studies (Bede & Hardiman, 2018; Menke et al., 2014; van der Graaff et al., 2011; Zhang et al., 2011). Histologically, inflammatory markers are increased in the corpus callosum with accompanying loss of myelinated axons (Cardenas et al., 2017; Sugiyama et al., 2013), although its role in the pathophysiology of the disease is yet to be elucidated.

In attempting to delineate the relationship between cerebral degeneration and the disease progression rate, patients with fast progressing ALS had greater longitudinal texture alterations in the precentral gyrus compared to patients with slow progressing ALS; however, at baseline, texture abnormalities were similar in both subgroups in this region. Pathology in the motor cortex likely approaches its maximal state, especially in patients with slower disease progression rates, by the time patients are enrolled in clinical studies. In support of this, several studies show correlations between disease progression rate and extra‐motor frontotemporal regions in whole‐brain cortical thickness analyses (d'Ambrosio et al., 2014; Verstraete et al., 2012; Walhout et al., 2015). It can be postulated from these observations that a faster disease progression rate implicates a more rapid and greater involvement of the frontotemporal regions. Conversely in the PLIC, patients with slow progressing ALS had greater longitudinal texture change whereas patients with fast progressing ALS had greater abnormalities at baseline. The pyramidal tracts may play a more direct role in regulating disease progression with faster rates being associated with greater degeneration at baseline (Menke et al., 2012). This is further substantiated by our observation of greater clinical UMN dysfunction in the fast progressing ALS subgroup at baseline. To the best of our knowledge, no other study has formally investigated the impact of disease progression rate on the progressive degeneration of gray and white matter structures in ALS. It should be noted that even though some of these findings did not satisfy strict statistical significance thresholds, they are of clinical significance and warrant further investigation. If patients with slower disease progression rates do indeed continue to experience progressive pyramidal tract degeneration, this may be an interventional window for future therapies.

### 4.2 Texture of T1‐weighted images as a marker for cerebral degeneration in ALS

The most consistent texture abnormalities in ALS are in the motor cortex and in the regions of the pyramidal tracts. Loss of Betz cells (Lawyer Jr. & Netsky, 1953; Nihei, McKee, & Kowall, 1993), astrocytic gliosis (Kamo et al., 1987; Murayama, Inoue, Kawakami, Bouldin, & Suzuki, 1991), and aberrant TDP‐43 deposition (Brettschneider et al., 2013) are considered the core pathological features in the motor cortex in ALS. Decreased autocorrelation in the motor cortex in patients is likely related to some aggregate of these abnormalities. The derivation of autocorrelation is a function of voxel intensities and the likelihood of co‐occurring intensities in an image. Indeed, histological associations, particularly with markers of gliosis, using quantitative ex vivo MRI have shown that these pathological features impact T1 relaxation times in ALS (Meadowcroft et al., 2015). Recent studies have also suggested that T2 shortening in the motor cortex is caused by increased iron accumulation in the microglia (Kwan et al., 2012). Reduced gray matter density (Shen et al., 2016) and cortical thinning (Agosta et al., 2012) are well‐known neuroimaging correlates of ALS. It is reasonable to attribute decreased autocorrelation simply to cortical atrophy. However, we showed previously that reduced autocorrelation values in the cortex only partially overlap with reduced gray matter densities and also expand to other disease‐related regions (Ishaque, Mah, Seres, Luk, Eurich, et al., 2018). Texture abnormalities could provide insight into events preceding the later stages of degeneration such as cortical thinning.

Within the pyramidal tracts, loss of myelinated axons and altered myelin sheath morphology are classic pathologic findings in ALS (Lawyer Jr. & Netsky, 1953; Smith, 1960). Findings of myelin pallor on Luxol fast blue stains, suggesting myelin loss, are variable and are often present only in cases of marked UMN loss. A study investigating in vivo myelin content with myelin water fraction found that there were no abnormalities in ALS patients compared to controls (Kolind et al., 2013). Instead, the authors found increased intra‐ and extracellular water content that is potentially associated with edema secondary to neuroinflammatory processes. This is in concordance with evidence of widespread cerebral microglial activation in ALS found in neuroimaging (Turner et al., 2004) and histological studies (Kawamata, Akiyama, Yamada, & McGeer, 1992). Additionally, mild qualitative hyperintensities on T1‐weighted images (Kato et al., 1997) and increased T2 relaxation times and quantitative proton density in ALS (Ding et al., 2011) are thought to be caused by axonal damage leading to an increase in unbound water. Taken together, we postulate that the increase in autocorrelation observed in the pyramidal tracts in ALS is caused by neuroinflammatory processes involved in the breakdown of myelin content and axonal loss secondary to cortical neuronal degeneration instead of primary insults to myelin content. Indeed, texture abnormalities in the pyramidal tracts in ALS also correlate with abnormalities in DTI measures that suggest a secondary axonal degeneration process (Ishaque, Mah, Seres, Luk, Johnston, et al., 2018). This is in contrast with multiple sclerosis where increased texture heterogeneity in lesions and diffusely abnormal white matter was found to be more sensitive to myelin loss compared to axonal injury and inflammation (Zhang et al., 2013). It is therefore imperative that future studies disentangle the multifaceted causes of texture alterations in ALS with direct correlations to histological data to further understand the pathophysiology of the disease.

In this study, textural changes were evident in the medial temporal lobes at baseline and longitudinally. Hippocampal involvement in patients with ALS has been documented postmortem (Brettschneider et al., 2013) and more recently in neuroimaging studies (Christidi et al., 2018; Christidi et al., 2019). Neuropsychological studies have also indicated the presence of memory differences in this patient population (Woolley & Rush, 2017). Despite the emergence of these findings, the pathophysiology of involvement in the medial temporal lobe in ALS is not yet clearly defined. Previously we have shown that texture analysis is a sensitive marker for hippocampal degeneration in Alzheimer's disease (Luk et al., 2018). In addition, we have demonstrated the importance of the involvement in extra‐motor structures, particularly the frontotemporal lobes, in a prior study. It can therefore be hypothesized that texture changes seen here at baseline and longitudinally represent an inherent disease process in ALS; however, correlative studies with cognitive data and confirmatory analysis with postmortem studies is crucially needed.

Clinical–radiological associations have been sought extensively in ALS (Verstraete et al., 2015). The near‐exclusive correlation between pyramidal tract changes and UMN burden in this study suggests that the degeneration of this pathway is primarily responsible for the clinical presentation of UMN dysfunction. This was similarly observed in a previous DTI study (Menke et al., 2014). In contrast, the widespread cerebral correlations of ALSFRS‐R underscore its poor specificity for UMN function. Studies have shown correlations between ALSFRS‐R limb and bulbar sub‐scores and the respective gray matter regions in the motor homunculus (Bede et al., 2013a; Walhout et al., 2015). Abnormalities in ALSFRS‐R sub‐scores also correlate with the region of symptom onset (Rooney, Burke, Vajda, Heverin, & Hardiman, 2017). The region of symptom onset in ALS is believed to experience the maximal UMN and LMN degeneration (Ravits, 2014). Therefore, ALSFRS‐R correlations along the motor homunculus are likely related to an interplay between concurrent UMN and LMN pathology. Finger and foot tapping scores have been used as surrogates for UMN dysfunction in ALS studies (Kent‐Braun, Walker, Weiner, & Miller, 1998; Mitsumoto et al., 2007). Here, we found these scores to correlate with clinical UMN dysfunction and as such demonstrate their sensitivity to it; however, they did not show specificity to motor cortex or pyramidal tract degeneration in whole‐brain correlations. Functional MRI studies have demonstrated reduced cortical activity in the prefrontal cortices during voluntary movement tasks in ALS patients (Cosottini et al., 2012; Stanton et al., 2007). This is in line with our finding of frontal lobe associations of finger and foot tapping scores and suggests that motor weakness related to volitional tasks in ALS is associated with failures and compensations in larger networks and not isolated dysfunctions in the UMN system.

### 4.3 Technical considerations and limitations

We have successfully shown here that 3D texture analysis of T1‐weighted images enables the assessment of gray and white matter structures for degenerative changes. These changes are associated with clinical impairment and can offer insight into the pathophysiology of disease and serve as markers in clinical trials. This technique represents an advancement for neuroimaging studies as it can interrogate both gray and white matter without requiring lengthy multimodal MRI protocols that are often challenging for patients with debilitating diseases. Texture features may therefore be considered robust markers for cerebral degeneration that can be rapidly implemented in clinical trials as they only require a T1‐weighted image. Future studies must investigate the neuropathological underpinnings for texture features and associations with other relevant MRI modalities (Ishaque, Mah, Seres, Luk, Johnston, et al., 2018).

We included longitudinal data from controls to ensure the observed findings were not due to healthy aging, or an artifact of texture analysis. A limitation of this study, however, is that since CALSNIC is actively acquiring data, longer duration, and complete follow‐up data were not available for all patients and controls. A future study with a larger dataset should aim to replicate the current findings. Furthermore, we did not apply image intensity normalization techniques in this study to account for multicentre data from different MRI systems. It is possible that some of the observed results were affected by MRI system‐specific differences. However, given the relevant clinical correlations, similar results to previous studies, and inclusion of control data from all recruiting sites, that is an unlikely possibility. Additionally, autocorrelation has demonstrated high intra‐ and intersite reliability in traveling control datasets (Ta et al., 2019). Nevertheless, it would be worthwhile to optimize texture analysis pipelines to account for possible subtle image intensity variations due to MRI system differences. Another limitation of this study is the lack of complete genetic information for all participants. Previous studies have examined the relationship between positive C9ORF expansion mutation and dissemination of cerebral pathology, particularly in frontotemporal structures (Hi et al., 2021). A future study should aim to evaluate and compare the spatiotemporal patterns of degeneration within genotypes of ALS using texture analysis. Lastly, the focus of this study was to evaluate variability in cerebral degeneration in phenotypes relating to rates of disease progression. However, cognitive and behavioral impairments have been well‐established as part of the clinical spectrum within ALS and contribute to disease heterogeneity. Future studies should explore the relationship between cognitive impairment and frontotemporal degeneration ascertained by 3D texture analysis.

In conclusion, we provide evidence for progressive degeneration of white matter in the PLIC in ALS over 4‐ and 8‐month intervals in the absence of clinical UMN decline. The longitudinal course of gray matter pathology is characterized predominantly by a frontotemporal spatial spread, instead of progressive degeneration within the motor structures. Furthermore, these progressive patterns are influenced by disease progression rate. This suggests the presence of disease‐specific cerebral network vulnerabilities and differential involvement of gray and white matter degeneration in contrast to a simple gradient. Indeed, future studies should look to further parse the factors influencing longitudinal degeneration in ALS, such as site of symptom onset and cognitive involvement.

## CONFLICT OF INTEREST

The authors report no competing financial and nonfinancial interests in relation to the work described.

## ETHICS STATEMENT

Institutional ethics approvals were obtained from all recruiting centers participating in the study.

## PATIENT CONSENT STATEMENT

All participants provided free and informed written consent prior to their involvement in the study.

## Supporting information


**Figure S1** Supporting information

**Table S1** Supporting information

## Data Availability

Data can be made available upon submission of a formal request to the corresponding author.
