# Supplementary material for: Distinct patterns of progressive gray and white matter degeneration in amyotrophic lateral sclerosis
Source: Hum Brain Mapp. 2021 Dec 15;43(5):1519–34. doi: 10.1002/hbm.25738 (PMC8886653; doi:10.1002/hbm.25738)
Supplement: Supplementary file 1 — Figure S1 Supporting information [file HBM-43-1519-s001.pdf]

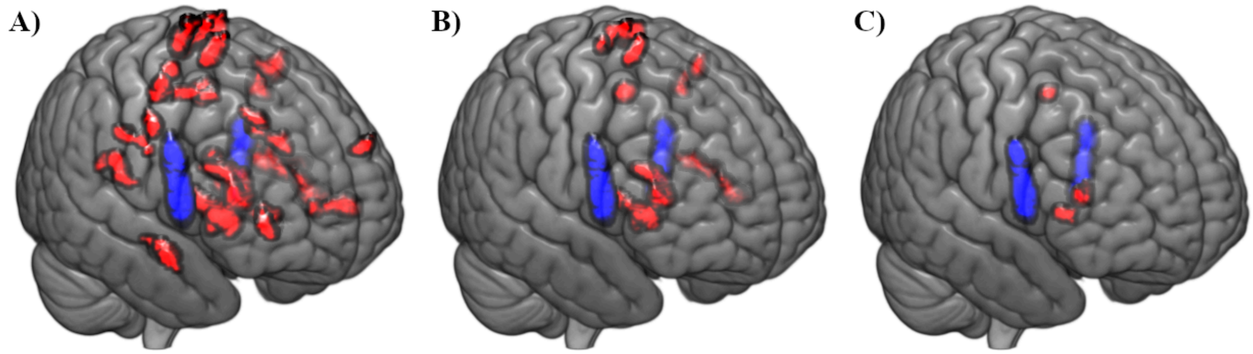

$P > 0.00005$ $P > 0.000005$ $P > 0.0000005$

Supplementary Figure 1: *Glass brain representations of group differences in texture between controls and ALS patients at different statistical thresholds. Red regions indicate decreased autocorrelation and blue regions indicate increased autocorrelation in ALS patients. At the lowest threshold (A), diffuse gray matter regions showed altered autocorrelation, including left frontal white matter, bilateral temporal white matter, cingulate gyrus, and thalamic region. At the intermediate threshold (B), left lateral precentral gyrus and left insular cortex demonstrated differences in autocorrelation between the two groups. At the highest statistical threshold (C), differences were found in the left medial precentral gyrus. Bilateral pyramidal tracts had increased autocorrelation at all statistical thresholds.*

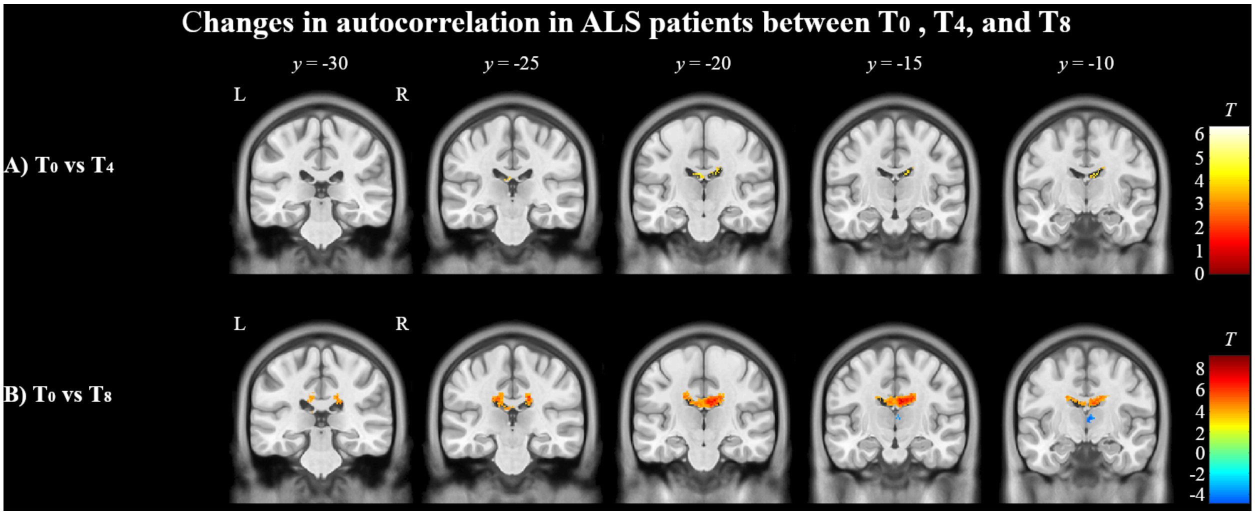

Supplementary Figure 2: *Longitudinal changes in texture in ALS between T_0, T_4, and T_8 ($P < 0.0005$, cluster size > 50). (A) Between T_0 and T_4, autocorrelation decreased in the posterior corpus callosum in ALS patients. Autocorrelation did not increase between these timepoints. (B) Between T_0 and T_8, autocorrelation decreased in the posterior corpus callosum and at the junction of the lateral ventricles and bilateral caudate heads. Autocorrelation also increased in the right thalamus. The color bars show the range of T-values.*

A Correlation between ALSFRS-R and UMN burden

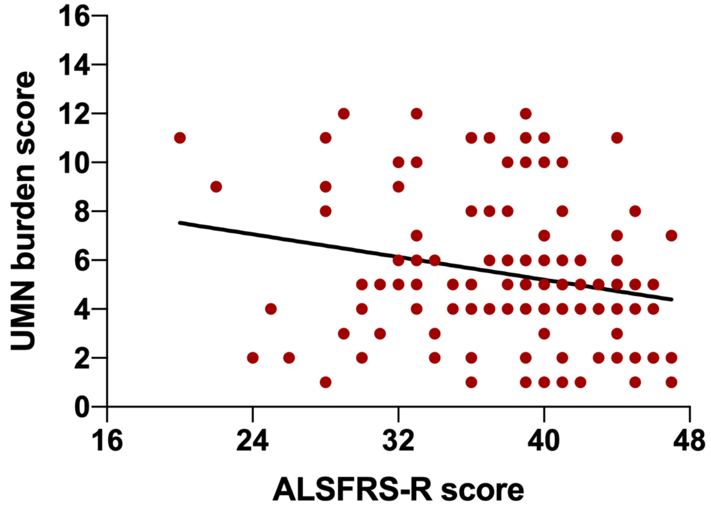

B Correlation between ALSFRS-R and finger tapping

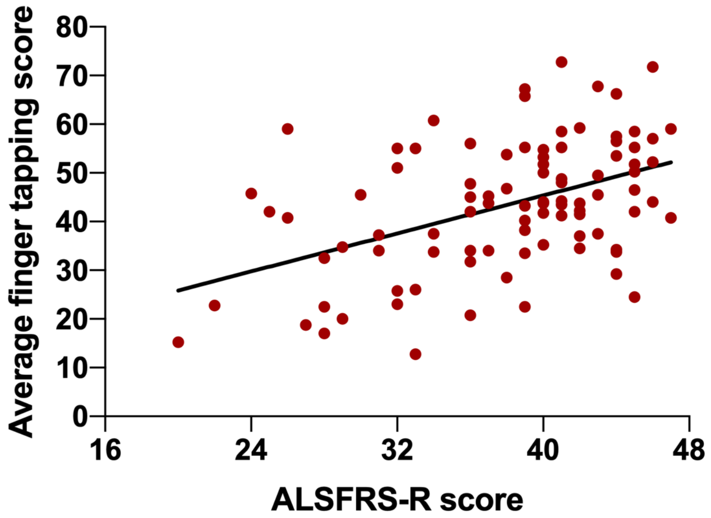

C Correlation between ALSFRS-R and foot tapping

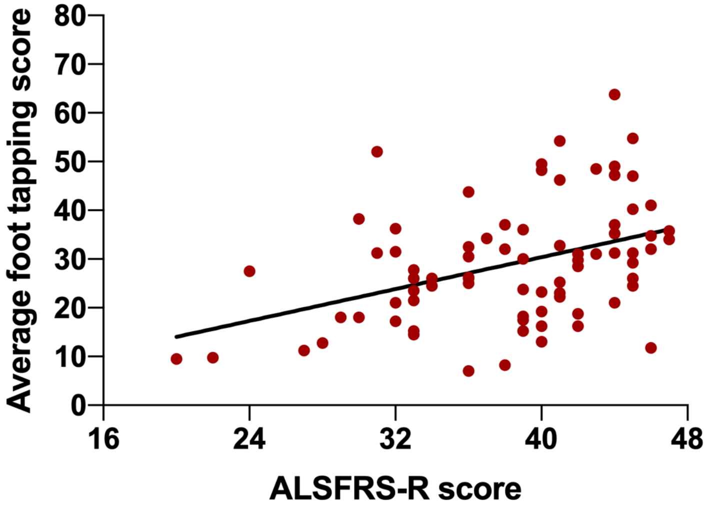

D Correlation between UMN burden and finger tapping

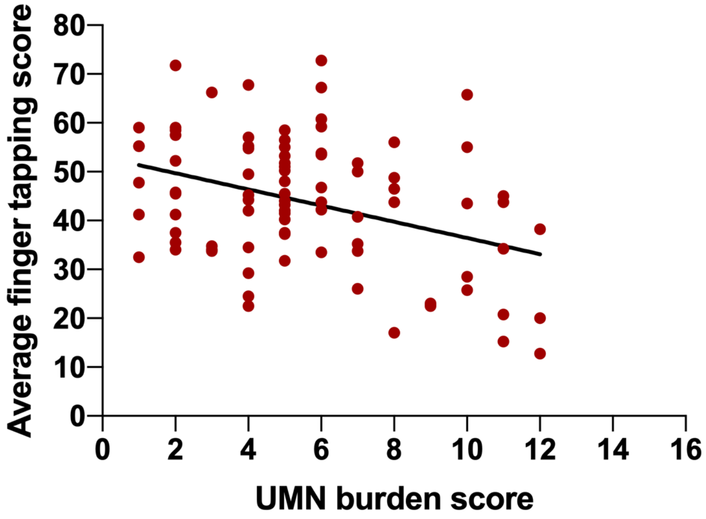

E

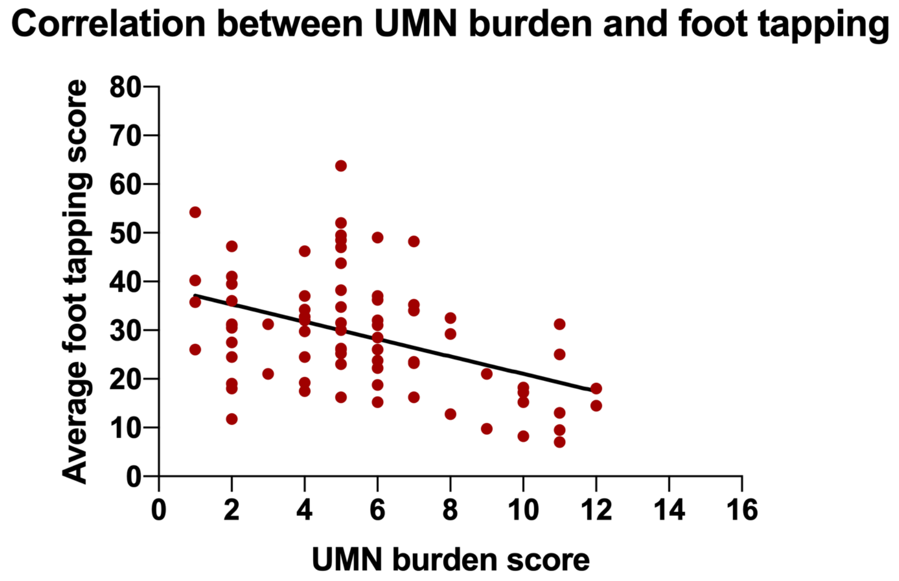

Supplementary Figure 3: *Correlations between clinical measures at baseline in ALS patients. ALSFRS-R score significantly correlated with (A) UMN burden score (r = -0.2, P = 0.01), (B) average finger tapping score (r = 0.5, P < 0.001), and (C) average foot tapping score (r = 0.4, P < 0.001). UMN burden score significantly correlated with (D) average finger tapping score (r = -0.4, P < 0.001) and (E) average foot tapping score (r = -0.4, P < 0.001).*

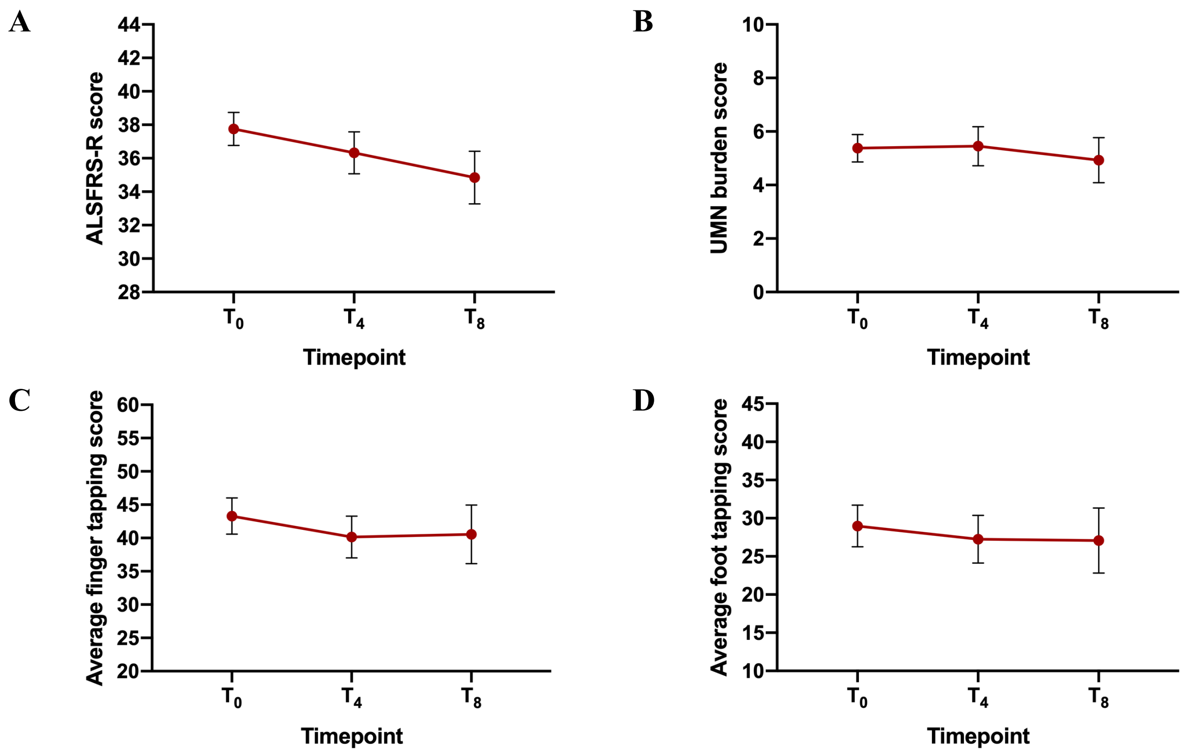

Supplementary Figure 4: *Clinical measures of all ALS patients at T_0, T_4, and T_8. Data is represented as the mean ± 95% confidence interval at each timepoint.*

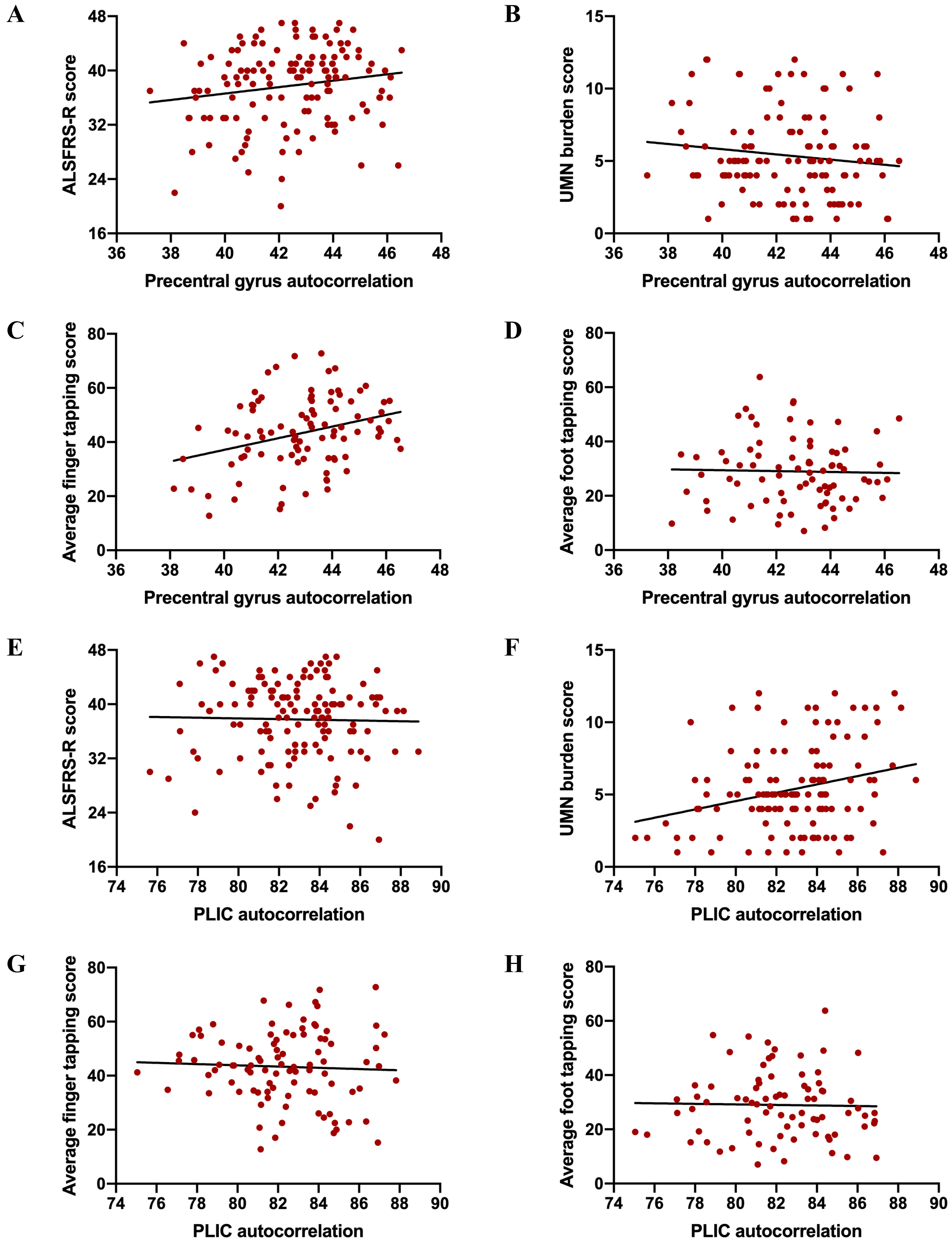

Supplementary Figure 5: *Associations between texture of the precentral and the posterior limb of the internal capsule (PLIC) and clinical measures in ALS patients. Significant correlations (P < 0.05) were found between autocorrelation of the precentral gyrus and average finger tapping score (C, r = 0.3). Autocorrelation of the PLIC correlated significantly with UMN burden score (F, r = 0.2).*
