# Supplementary material for: Distinct patterns of progressive gray and white matter degeneration in amyotrophic lateral sclerosis
Source: Hum Brain Mapp. 2021 Dec 15;43(5):1519–34. doi: 10.1002/hbm.25738 (PMC8886653; doi:10.1002/hbm.25738)
Supplement: Supplementary file 2 — Table S1 Supporting information [file HBM-43-1519-s002.pdf]

Supplementary Table 1: *Calculation of the upper motor neuron burden score. Muscle tone and limb reflexes were tested bilaterally in arms and legs. The score associated with the presence of the clinical sign is provided in brackets.*

Clinical exam	Neurological sign and score
Muscle tone (x4)	Hypertonia (1)
Reflexes	
Jaw reflex	Hyperreflexia (1), clonus (2)
Limb reflexes (x4)	Hyperreflexia (1), clonus (2)
Babinski reflex (x2)	Positive (1)
Total UMN burden score	/16

UMN = upper motor neuron

Supplementary Table 2: *MRI acquisition parameters for 3D T1-weighted scans (all 1 mm isotropic) at the six centres included in this study. Most centres implemented two different protocols as part of the Canadian ALS Neuroimaging Consortium.*

Centres	University of British Columbia	University of Calgary		University of Alberta	
	Protocol 1	Protocol 1	Protocol 2	Protocol 1	Protocol 2
n (controls/patients)	10/9	9/9	11/7	19/17	26/21
Scanner vendor	Philips	General Electric	General Electric	Siemens	Siemens
Head coil channels	8	12	32	20	64
Acquisition matrix	240 x 240	256 x 256	224 x 256	256 x 256	232 x 256
Field of view (mm^2)	240 x 240	256 x 256	256 x 256	256 x 256	232 x 256
Slice thickness (*mm*)	1	1	1	1	1
Orientation	Axial	Axial	Sagittal	Axial	Sagittal
Number of slices	150	176	176	176	176
Repetition time (*ms*)	7.9	7.4	8.1	2300	1700
Echo time (*ms*)	3.5	3.1	3.2	3.4	2.2
Inversion time (*ms*)	950	400	400	900	880
Flip angle (°)	8	11	16	9	10

Centres	University of Toronto		McGill University		Laval University
	Protocol 1	Protocol 2	Protocol 1	Protocol 2	Protocol 1
n (controls/patients)	11/23	8/20	6/12	10/8	9/11
Scanner vendor	General Electric	Siemens	Siemens	Siemens	Philips
Head coil channels	8	64	32	64	8
Acquisition matrix	256 x 256	232 x 256	256 x 256	232 x 256	256 x 256
Field of view (mm^2)	256 x 256	232 x 256	256 x 256	232 x 256	256 x 256
Slice thickness (*mm*)	1	1	1	1	1
Orientation	Axial	Sagittal	Axial	Sagittal	Sagittal
Number of slices	176	176	176	176	176
Repetition time (*ms*)	7.7	1700	2300	1700	7.1
Echo time (*ms*)	2.9	2.2	3.4	2.2	3.4
Inversion time (*ms*)	400	880	900	880	950
Flip angle (°)	11	10	9	10	10

Supplementary Table 3: *Characteristics of slow and fast progressing ALS patients at baseline. Data is represented as mean ± standard deviation. Significant between-group differences (P < 0.05) are highlighted in bold.*

	Slow progressing ALS	Fast progressing ALS	*P* value
n	67	67	
Age (years)	59.7 ± 11.1	58.2 ± 9.2	0.4[a]
Gender, male/female	44/23	40/27	0.5[b]
Site of symptom onset, limb/bulbar	61/6	47/19	**0.004[b]**
ALSFRS-R	40.0 ± 4.9	35.5 ± 5.7	**< 0.001[a]**
Symptom duration (months)	45.2 ± 28.8	19.5 ± 11.2	**< 0.001[a]**
Disease progression rate	0.2 ± 0.09	0.7 ± 0.4	**< 0.001[a]**
UMN burden score	4.6 ± 2.5	6.3 ± 3.0	**0.002[a]**
Finger tapping score	46.6 ± 12.2	40.6 ± 13.8	**0.03[a]**
Foot tapping score	31.2 ± 11.7	26.9 ± 12.6	0.1[a]

UMN = upper motor neuron; LMN = lower motor neuron; ALSFRS-R = amyotrophic lateral sclerosis functional rating scale-revised; disease progression rate = (48 – ALSFRS-R)/symptom duration; $\Delta T_{0\text{-}max}$ = time interval between baseline and last follow-up MRI; [a] = independent samples *t*-test; [b] = chi-squared test
